# Sensitive Troponin I Assay in Patients with Chest Pain - Association with Significant Coronary Lesions with or Without Renal Failure

**DOI:** 10.5935/abc.20170182

**Published:** 2018-01

**Authors:** Alexandre de Matos Soeiro, Danielle Menosi Gualandro, Aline Siqueira Bossa, Cindel Nogueira Zullino, Bruno Biselli, Maria Carolina Feres de Almeida Soeiro, Tatiana de Carvalho Andreucci Torres Leal, Carlos Vicente Serrano Jr., Mucio Tavares de Oliveira Junior

**Affiliations:** Unidade Clínica de Emergência - InCor - HCFMUSP, São Paulo, SP - Brazil

**Keywords:** Troponin I, Chest Pain, Coronary Artery Disease, Renal Insufficiency, Chronic, Biomarkers

## Abstract

**Introduction:**

Despite having higher sensitivity as compared to conventional troponins, sensitive troponins have lower specificity, mainly in patients with renal failure.

**Objective:**

Study aimed at assessing the sensitive troponin I levels in patients with chest pain, and relating them to the existence of significant coronary lesions.

**Methods:**

Retrospective, single-center, observational. This study included 991 patients divided into two groups: with (N = 681) and without (N = 310) significant coronary lesion. For posterior analysis, the patients were divided into two other groups: with (N = 184) and without (N = 807) chronic renal failure. The commercial ADVIA Centaur^®^ TnI-Ultra assay (Siemens Healthcare Diagnostics) was used. The ROC curve analysis was performed to identify the sensitivity and specificity of the best cutoff point of troponin as a discriminator of the probability of significant coronary lesion. The associations were considered significant when p < 0.05.

**Results:**

The median age was 63 years, and 52% of the patients were of the male sex. The area under the ROC curve between the troponin levels and significant coronary lesions was 0.685 (95% CI: 0.65 - 0.72). In patients with or without renal failure, the areas under the ROC curve were 0.703 (95% CI: 0.66 - 0.74) and 0.608 (95% CI: 0.52 - 0.70), respectively. The best cutoff points to discriminate the presence of significant coronary lesion were: in the general population, 0.605 ng/dL (sensitivity, 63.4%; specificity, 67%); in patients without renal failure, 0.605 ng/dL (sensitivity, 62.7%; specificity, 71%); and in patients with chronic renal failure, 0.515 ng/dL (sensitivity, 80.6%; specificity, 42%).

**Conclusion:**

In patients with chest pain, sensitive troponin I showed a good correlation with significant coronary lesions when its level was greater than 0.605 ng/dL. In patients with chronic renal failure, a significant decrease in specificity was observed in the correlation of troponin levels and severe coronary lesions.

## Introduction

In recent years, cardiology has witnessed the constant development of several biomarkers, of which, current sensitive troponins and high-sensitivity troponins, widespread in Brazil and Europe, stand out.^1^

However, despite the huge gain in sensitivity, allowing early detection of a minimum threshold of myocardial lesion in patients presenting to the emergency department with chest pain, there was a reduction in specificity, which resulted in several patients with non-cardiological or non-coronary problems undergoing unnecessary and even harmful antithrombotic therapy and invasive coronary stratification.^2-5^ The adequate troponin level to be considered for the correct interpretation of clinical findings depends on the patient’s characteristics and on the troponin assay used, and should be ideally individualized for each service.^2-4,6^

Thus, this study was aimed at assessing the current sensitive troponin I levels for patients with chest pain, in addition to relating them to the existence of significant coronary lesions both in the presence and absence of chronic renal failure in the sample selected.

## Methods

### Study population

This is a retrospective, single-center, observational study, including 991 patients with chest pain admitted to the emergency department of a high-complexity tertiary cardiology center, between May 2013 and May 2015.

All patients with chest pain undergoing coronary angiography for suspected unstable angina or non-ST-elevation acute myocardial infarction were included. Presence of ST-segment elevation was the only exclusion criterion. The coronary lesion was considered significant when ≥ 70% on coronary angiography. Chronic renal failure was defined as a creatinine level > 1.5 mg/dL.

The patients were divided into two groups: with (N = 681) and without (N = 310) significant coronary lesion. For Receiver Operating Characteristic (ROC) curve analysis, the patients were divided into two other groups: with (N = 184) and without (N = 807) chronic renal failure.

The commercial ADVIA Centaur^®^ TnI-Ultra assay (Siemens Healthcare Diagnostics, Tarrytown, NY, USA) was used for current sensitive troponin with a 99^th^ percentile value of 0.04 ng/mL. The flowchart of the management of all patients with chest pain met the criteria established by the last American Heart Association guideline.^7-9^ Non-ST-elevation acute coronary syndrome was defined as presence of chest pain associated with electrocardiographic changes or troponin elevation/drop on admission or, in the lack thereof, clinical findings and risk factors compatible with unstable angina (chest pain at rest or on minimal exertion, of severe intensity or occurring in a *crescendo* pattern). The highest troponin level during hospitalization before coronary angiography was considered for analysis, following the every 6-hour marker collection protocol of the institution.

The following data were obtained: age, sex, presence of diabetes mellitus, systemic arterial hypertension, smoking habit, dyslipidemia, family history of early coronary artery disease, chronic coronary artery disease, previous acute myocardial infarction, creatinine, ST-segment depression or T-wave inversion on the electrocardiogram.

This study was submitted to the Ethics Committee in Research and approved by it. All patients provided written informed consent.

### Statistical analysis

The ROC curve analysis was performed to identify the sensitivity and specificity of the best cutoff point of troponin as a discriminator of the probability of significant coronary lesion, and 95% confidence interval (CI) was used. That analysis was performed for the general population and separately for patients with and without chronic renal failure.

Descriptive analysis of the categorical variables was performed by use of percentages. Continuous variables with non-normal distribution were expressed as medians and interquartile intervals, and those with normal distribution, as means and standard deviations. The comparison between groups was performed by use of the chi-square test for categorical variables. The continuous variables, when the Kolmogorov-Smirnov test showed normal distribution, were assessed by using the unpaired T test, and when the distribution was not normal, the Mann-Whitney U test was used. Both troponin cutoff points analyzed (the 99^th^ percentile of the method and the best cutoff point found in this study) were entered into the univariate analysis. Comparison between patients with *versus* without significant coronary lesion was performed.

Multivariate analysis was performed with logistic regression, p < 0.05 being the significance level adopted. All baseline characteristics listed in Table 1 that reached statistical significance on univariate analysis were considered as variables in the analysis. Multivariate analysis was performed separately for each troponin cutoff point assessed (the 99^th^ percentile of the method and the best cutoff point found in this study).

**Table 1:** Baseline characteristics and univariate analysis comparing patients with versus without significant coronary lesion

	Coronary lesions ≥ 70%		p
	Present (N = 681)	Absent (N = 310)	
Male sex (%)	72.10%	65.10%	0.018^#^
Age (median)	62.9 ± 11.30	63.9 ± 13.23	0.202^π^
Diabetes mellitus (%)	38.82%	40%	0.725^#^
Arterial hypertension (%)	79.30%	84.80%	0.038^#^
Chronic coronary disease (%)	13.70%	14.50%	0.724^#^
Dyslipidemia (%)	51.00%	50.00%	0.797^#^
FH of early CAD (%)	12.50%	10.60%	0.404^#^
Previous AMI (%)	39.70%	36.10%	0.284^#^
Smoking (%)	43.50%	31.30%	< 0.0001^#^
Creatinine (mg/dL) (mean)	1.31 ± 1.20	1.32 ± 1.25	0.896*
ST depression/T-wave inversion	36.30%	18.70%	< 0.0001^#^
Troponin + / 99^th^ percentile	91.50%	72.60%	< 0.0001^#^
Troponin + / Best cutoff point	63.40%	32.60%	< 0.0001^#^

FH: family history; CAD: coronary artery disease; AMI: acute myocardial infarction;

# chi square test;

* unpaired T test;

π Mann-Whitney U test.

The calculations were performed with the SPSS software, version 10.0.

## Results

**Figure 1:**
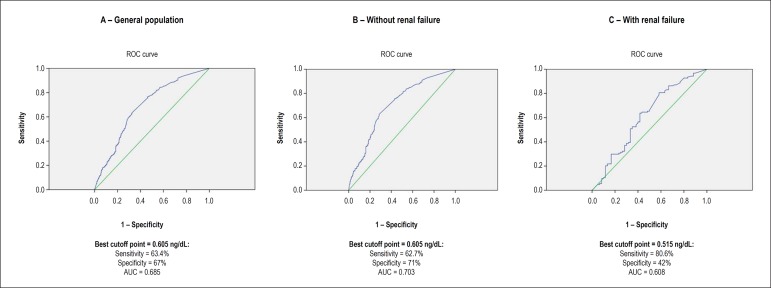
ROC curve identifying the sensitivity and the specificity of the best cutoff point of troponin as a discriminator of the probability of significant coronary lesion. AUC: area under the curve.

The median age was 63 years, and 52% of the patients were of the male sex. The area under the ROC curve between the troponin levels and significant coronary lesions was 0.685 (95% CI: 0.65 - 0.72). In patients with or without renal failure, the areas under the ROC curve were 0.703 (95% CI: 0.66 - 0.74) and 0.608 (95% CI: 0.52 - 0.70), respectively. The best cutoff points to discriminate the presence of significant coronary lesion were: in the general population, 0.605 ng/dL (sensitivity, 63.4%; specificity, 67%; positive predictive value, 65.9%; negative predictive value, 64.7%; accuracy, 65.3%; and likelihood ratio, 1.9); in patients without renal failure, 0.605 ng/dL (sensitivity, 62.7%; specificity, 71%; accuracy, 66.9%; and likelihood ratio, 2.2); and in patients with chronic renal failure, 0.515 ng/dL (sensitivity, 80.6%; specificity, 42%; accuracy, 61.3%; and likelihood ratio, 1.4) (Figure 1). In the general population, the level of 0.05 ng/dL (immediately above the 99^th^ percentile) showed sensitivity of 93.7% and specificity of 23%. For patients with chronic renal failure to reach a specificity of 67% (as in the general population), an elevation in the troponin level to 1.58 ng/dL was necessary.

Troponin was negative in 143 patients, and, in 40.6% of them, significant lesions were observed on coronary angiography. In addition, 10.5% of those patients with negative troponin showed ST-segment depression/T-wave inversion on electrocardiogram. Using the gold-standard procedure of cardiac catheterization, the acute coronary syndrome diagnosis was confirmed in 68.7% of the patients admitted due to chest pain. In 9.1% of those without significant coronary lesion on coronary angiography and with positive troponin, the acute coronary syndrome diagnosis was confirmed by cardiac magnetic resonance. The baseline characteristics of the population studied and the univariate analysis between the groups are shown in Table 1.

In multivariate analysis, considering the 99^th^ percentile of the method, there were significant differences between the groups with and without coronary lesion regarding smoking habit (OR = 1.58, p = 0.002), ST-segment depression/T-wave inversion (OR = 2.05, p < 0.0001) and troponin positivity (OR = 3.39, p < 0.0001), respectively. However, when considering the best troponin cutoff point found in this study, there were significant differences between the groups with and without coronary lesion regarding the male sex (OR = 1.35, p = 0.039), smoking habit (OR = 1.64, p = 0.001), ST-segment depression/T-wave inversion (OR = 2.22, p < 0.0001) and troponin positivity (OR = 3.39, p < 0.0001), respectively. The multivariate analysis results are shown in Table 2.

**Table 2:** Multivariate analysis comparing patients with *versus* without significant coronary lesion: A. Using the 99^th^ percentile of the troponin assay; B. using the best cutoff point for troponin found in the study

**A**			
	**OR**	**95% CI**	**p**
Male sex (%)	1.32	0.99 - 1.76	0.052
Arterial hypertension (%)	0.81	0.55 - 1.18	0.272
Smoking (%)	1.58	1.18 - 2.14	0.002
ST depression/T-wave inversion	2.05	1.47 - 2.88	< 0.0001
Troponin + / 99^th^ percentile	3.39	2.32 - 4.94	< 0.0001
			
**B**			
	**OR**	**95% CI**	**p**
Male sex (%)	1.35	1.02 - 0.180	0.039
Arterial hypertension (%)	0.89	0.60 - 1.31	0.548
Smoking (%)	1.64	1.21 - 2.22	0.001
ST depression/T-wave inversion	2.22	1.58 - 3.12	< 0.0001
Troponin + / Best cutoff point	3.39	2.53 - 4.54	< 0.0001

OR: odds ratio; CI: confidence interval.

## Discussion

The results of this study in the Brazilian population are in accordance with those of recently published literature. Troponin positivity without association with coronary angiographic findings was observed in 31.3% of the patients. In addition, better specificity values were only achieved with a troponin cutoff point of 0.605 ng/dL, approximately 15 times the 99^th^ percentile of the method. When assessing the subgroup with renal failure, that level is even higher, hindering its correct interpretation.

In a study published in 2012 derived from the Scottish Heart Health Extended Cohort, blood samples were collected and high-sensitivity troponin I levels were measured. The results showed that, in a population of 15340 individuals, 31.7% of the men and 18.1% of the women had high high-sensitivity troponin with no clinical manifestation at the time of blood collection, highlighting the problem of the specificity of the method. Positivity and worse prognosis were correlated in the long run (p < 0.0001), as reported in other studies.^4,10-12^ That prevalence of troponin positivity not related to acute coronary artery disease is similar to that found in our study, although we assessed specifically patients with chest pain.

Likewise, a prospective cohort study of 6304 patients with chest pain presenting to the emergency department has reported positive high-sensitivity troponin T in 39% of the cases diagnosed as non-coronary.^13^

Irfan et al.^14^ have conducted an observational multicenter study with 1181 patients hospitalized because of non-cardiac causes, 15% of whom had positive high-sensitivity troponin T. Of the major factors related to that unexpected elevation, the presence of kidney dysfunction was identified as a significantly influencing factor. In addition, once again, patients with elevated troponin were at higher risk for death (HR = 3.0; p = 0.02).^14^

In individuals older than 75 years, high-sensitivity troponin T was assessed in the context of chest pain, being measured at baseline and 3-4 hours. Approximately 27% of the patients were classified as having acute coronary syndrome. The sensitivity and specificity found in that population were 88% and 38%, respectively. The greater the initial level or the increase (mainly absolute) in the subsequent measures, the higher the specificity found.^15^ That specificity value can be greater than ours found in the general population, probably because of the inclusion of more patients with other heart diseases, because we belong to a referral tertiary cardiology center.

The concept of variation in the levels of sensitive troponin and high-sensitivity troponin in different measurements has been studied, and establishing a correlation between the amplitude of variability and the probability of coronary artery disease has been consecutively attempted. In addition, amplitude can be relative (expressed as percentages) or absolute, with possible implications and distinct interpretations.^1^

A retrospective study published in 2014, including 1054 patients with chest pain, assessed the variability related to high-sensitivity troponin T. Approximately 40% of the patients showed alteration in at least one measurement. Even with a variation greater than 20% as compared to the initial level, the specificity did not exceed 70%.^16^

Assessing specifically the same current sensitive troponin assay used in this study, in 2013 Bonaca et al.^17^ published a study comparing current sensitive troponin I *versus* high-sensitivity troponin I in 381 patients with chest pain at the emergency department. Those authors found sensitivity values for the two assays of 94% and 97%, and negative predictive values of 98% and 99%, respectively, with no significant difference.^17^ Another similar study of 1807 patients with non-ST-segment elevation acute coronary syndrome has shown no significant difference regarding prognosis when comparing the positivity of current sensitive troponin I *versus* high-sensitivity troponin I.^18^ Differently from the findings of those studies and using the same assay, ours showed lower sensitivity and specificity of 23% when using the 99^th^ percentile of the method. That shows the importance of assessing each center’s population, respecting their specific individualities.

In alignment with that, the meta-analysis published in 2014 with 17 studies and 8644 patients with chest pain compared the use of high-sensitivity troponin with that of conventional troponin. There were differences regarding sensitivity (88.4% vs. 74.9%; p < 0.001) and specificity (81.6% vs. 93.8%; p < 0.001), respectively. Despite that increase in sensitivity with high-sensitivity troponin, the number of patients with the final diagnosis of myocardial infarction and the need for additional tests for ischemia did not differ between the groups, showing no additional clinical advantage with the use of high-sensitivity troponin.^2^

Finally, some studies have validated the new troponin assays.^1,19,20^ The study conducted in 2015 compared seven assays of current sensitive troponins and high-sensitivity troponin in 2813 patients with chest pain, and with (16%) or without kidney dysfunction. Of the patients with nephropathy, in only 45-80% of those with positive troponin, the final diagnosis was myocardial infarction. The optimal cutoff point varied from 1.9 to 3.4 times that of the general population to detect acute coronary artery disease. Assessing only the same current sensitive troponin assay used in this study, in 27% of those with positive troponin, the final diagnosis of myocardial infarction was ruled out. The area under the curve of accuracy of that assay decreased from 0.92 to 0.87 (p = 0.013), comparing the general population with the patients with kidney dysfunction.^19^ That cutoff point elevation is in accordance with our findings, showing a clear specificity reduction in the group of patients with nephropathy.

### Limitations

Despite the large case series, this is a retrospective (hindering the blinded analysis) single-center study, with a much higher number of patients without chronic renal failure than with it. In addition, we used only one troponin assay, and most patients were of the male sex.

## Conclusion

In the study population of patients with chest pain, sensitive troponin I showed a good correlation with significant coronary lesions when its level was greater than 0.605 ng/dL. In patients with chronic renal failure, a significant decrease in specificity was observed in the correlation of troponin levels and severe coronary lesions.
